# The economic impact of endemic respiratory disease in pigs and related interventions - a systematic review

**DOI:** 10.1186/s40813-023-00342-w

**Published:** 2023-10-17

**Authors:** Marloes Boeters, Beatriz Garcia-Morante, Gerdien van Schaik, Joaquim Segalés, Jonathan Rushton, Wilma Steeneveld

**Affiliations:** 1 Department of Population Health Sciences, section Farm Animal Health, Faculty of Veterinary Medicine, Utrecht University, Utrecht, the Netherlands; 2 IRTA. Programa de Sanitat Animal. Centre de Recerca en Sanitat Animal (CReSA), Universitat Autònoma de Barcelona (UAB), Campus, Bellaterra, Catalonia 08193 Spain; 3 WOAH Collaborating Centre for the Research and Control of Emerging and Re-Emerging Swine Diseases in Europe (IRTA-CReSA), Bellaterra, 08193 Spain; 4 Unitat Mixta d’Investigació IRTA-UAB en Sanitat Animal, Centre de Recerca en Sanitat Animal (CReSA), Campus de la Universitat Autònoma de Barcelona (UAB), Bellaterra, 08193 Spain; 5 Royal GD, Deventer, the Netherlands; 6 Departament de Sanitat i Anatomia Animals, Facultat de Veterinària, Universitat Autònoma de Barcelona, Bellaterra, 08193 Spain; 7 Institute of Infection, Veterinary and Ecological Sciences, School of Health and Life Sciences, University of Liverpool, Liverpool, UK; 8 Global Burden of Animal Diseases (GBADs) Programme, University of Liverpool, Liverpool, UK

**Keywords:** Systematic review, Economic impact, Respiratory Disease, Pigs

## Abstract

**Background:**

Understanding the financial consequences of endemically prevalent pathogens within the porcine respiratory disease complex (PRDC) and the effects of interventions assists decision-making regarding disease prevention and control. The aim of this systematic review was to identify what economic studies have been carried out on infectious endemic respiratory disease in pigs, what methods are being used, and, when feasible, to identify the economic impacts of PRDC pathogens and the costs and benefits of interventions.

**Results:**

By following the PRISMA method, a total of 58 studies were deemed eligible for the purpose of this systematic review. Twenty-six studies used data derived from European countries, 18 from the US, 6 from Asia, 4 from Oceania, and 4 from other countries, i.e., Canada, Mexico, and Brazil. Main findings from selected publications were: (1) The studies mainly considered endemic scenarios on commercial fattening farms; (2) The porcine reproductive and respiratory syndrome virus was by far the most studied pathogen, followed by *Mycoplasma hyopneumoniae*, but the absence or presence of other endemic respiratory pathogens was often not verified or accounted for; (3) Most studies calculated the economic impact using primary production data, whereas twelve studies modelled the impact using secondary data only; (4) Seven different economic methods were applied across studies; (5) A large variation exists in the cost and revenue components considered in calculations, with feed costs and reduced carcass value included the most often; (6) The reported median economic impact of one or several co-existing respiratory pathogen(s) ranged from €1.70 to €8.90 per nursery pig, €2.30 to €15.35 per fattening pig, and €100 to €323 per sow per year; and (7) Vaccination was the most studied intervention, and the outcomes of all but three intervention-focused studies were neutral or positive.

**Conclusion:**

The outcomes and discussion from this systematic review provide insight into the studies, their methods, the advantages and limitations of the existing research, and the reported impacts from the endemic respiratory disease complex for pig production systems worldwide. Future research should improve the consistency and comparability of economic assessments by ensuring the inclusion of high impact cost and revenue components and expressing results similarly.

**Supplementary Information:**

The online version contains supplementary material available at 10.1186/s40813-023-00342-w.

## Background

Respiratory disease, referred to as the porcine respiratory disease complex (PRDC) when multiple pathogens and non-infectious factors are involved, is regarded as one of the most serious health problems in contemporary pig production. In Europe, pneumonia and pleuritis are the most frequent lung lesions observed at the slaughterhouse, with prevalence up to 69% and 48%, respectively [1–5]. In the United States, results from the 2012 National Animal Health Monitoring System indicated that respiratory problems were the main cause of deaths in weaned (47.3%) and grower/finisher pigs (75.1%) [6]. Besides increasing mortality, the PRDC is believed to induce production losses through reduced growth rates and a lower feed conversion efficiency [7, 8]. Consequently, respiratory disease remains one of the main reasons for antimicrobial usage in both nursery and growing/finishing pigs [9–11].

The PRDC term was coined to emphasise the complexity of events leading to respiratory disease development, including the involvement of (several) viral and bacterial pathogens as well as environmental, management, and genetic factors [12, 13]. Pathogens involved in the PRDC vary considerably in different countries, regions, and herds over time [14, 15]. The most common primary viral agents include porcine reproductive and respiratory syndrome virus (PRRSV), porcine circovirus 2 (PCV-2), and swine influenza virus (SIV) [12, 13, 16]. Other primary pathogens such as pseudorabies virus and porcine respiratory coronavirus are reported but they have less impact on today’s porcine health [17]. The bacterial species involved in this disease complex are traditionally distinguished between primary or initiators, such as *Mycoplasma* (*M.*) *hyopneumoniae*, and *Actinobacillus* (*A.*) *pleuropneumoniae*, and secondary or follower pathogens (e.g., *Pasteurella multocida*, *Streptococcus suis* and *Bordetella bronchiseptica*) [12, 13, 16]. The presence of various infectious agents in cases of PRDC leads to complex and potentially synergistic interactions that can increase the severity and duration of clinical signs and lesions, as well as the economic consequences [17].

As economic margins on pig farms are generally small [18], it is valuable to understand costs caused by endemically prevalent individual and co-existing pathogens within the PRDC, as there may be opportunities to increase farm profitability by controlling or preventing these infections. Therefore, estimates of costs and benefits of mitigation measures, can support decision-making regarding disease control at farm, integration system, regional and national levels.

Although one would expect the economic impact of respiratory disease to be well studied for the abovementioned reasons, no review or meta-analysis exists that maps the current state of economic research in this field. The economic implications of pathogens involved in the PRDC are likely to be heavily impacted by the variety in production systems and endemically prevalent strains of different pathogens across countries, as well as by the applied economic methods. These methods are defined by both the type of economic analysis (e.g. basic cost computations, partial budget analysis, cost-benefit analysis) and the cost components considered in this analysis (e.g. labour costs, feed costs, veterinary costs). Thus, the aim of this systematic review was to identify what economic studies have been carried out on infectious endemic respiratory disease in pigs, what economic methods are being used, and, when feasible, to identify the economic impacts of specific or co-existing PRDC pathogens and the costs and benefits of interventions.

## Materials and methods

A systematic literature review was conducted to identify relevant economic research on infectious endemic respiratory disease in pigs and related interventions. The Preferred Reporting Items for Systematic Reviews and Meta-Analyses (PRISMA 2020) guidelines were followed [19], without the use of risk-of-bias analysis (e.g. assessing the selection bias, reporting bias per study).

### Literature search

The search for suitable references was conducted in PubMed®, Scopus and CAB Abstracts. We restricted the search to studies published after January 1, 1980, and to peer-reviewed original research in English only. The search strings consisted of three parts (topic, population and focus), which were all required to be present in the title or abstract for a study to be included (for the full search strings, please refer to Supplementary file S1). The terms related to respiratory disease (topic) included terminology for both respiratory disease at syndrome level and for specific respiratory pathogens. The pathogens included were the most common infectious agents within the PRDC that are considered endemic in large parts of the world: the viral agents PRRSV, SIV and PCV-2, and the bacterial agents *M. hyopneumoniae* and *A. pleuropneumoniae*. The systematic search was lastly updated on January 23, 2023.

### Selection of studies

The abstracts obtained from the search were screened by two independent reviewers (co-authors MB and BGM). Studies were excluded when their main focus was not on respiratory disease in pigs and/or when no mention was made of an impact on either production parameters (e.g. average daily gain, mortality or feed conversion ratio) or on costs or revenues. The two reviewers compared and merged their findings and created a list of studies for the full text review, which was likewise carried out independently by MB and BGM. At this stage, only studies were included when the full text was available, when the report provided a full (e.g., farm budget analysis, cost-benefit analysis) or partial financial evaluation (e.g., cost analysis, basic calculation of medication costs), and when all calculated changes in economic outputs could be attributed to respiratory disease or to the interventions aiming to reduce or control the disease.

In addition, all open-access issues from the Journal of Swine Health and Production (JSHAP) were manually checked, as this journal is not indexed in a number of databases. Studies that met the screening and eligibility criteria were included. Lastly, reference lists and citations of all selected studies were examined for additional studies that met all inclusion criteria (literature snowballing).

### Data extraction

Data from the eligible studies were manually extracted by MB and BGM (not independently) and an online spreadsheet for data entry was used. These metadata included study characteristics related to publication (authors, year of publication, country and journal), study focus (syndrome or pathogen level, disease or intervention, unit of interest, farm type and animal age-group), study design (observational, experimental or simulation model) and economic methodology (type of economic evaluation, cost/revenue components, reported economic outcomes and currency). Additionally, we registered the origin of the data used in each study (e.g. primary data collected by the authors, expert opinion, data from scientific literature) and whether the paper mentions the testing of or accounting for the presence of other PRDC pathogens. All collected data are summarized in the text and provided in the Supplementary Files. Where we provide economic outcomes from the included studies, we adjusted the reported study outcomes for inflation using an online tool (https://in2013dollars.com/) and converted the original currency to euros using a currency converter tool (https://cuex.com/en/) (last applied on September 29, 2023). Where applicable, simple calculations were performed to reach a common unit to express the study results, such as the economic impact per fattening pig.

## Results

### Overview of the included studies

**Fig. 1:**
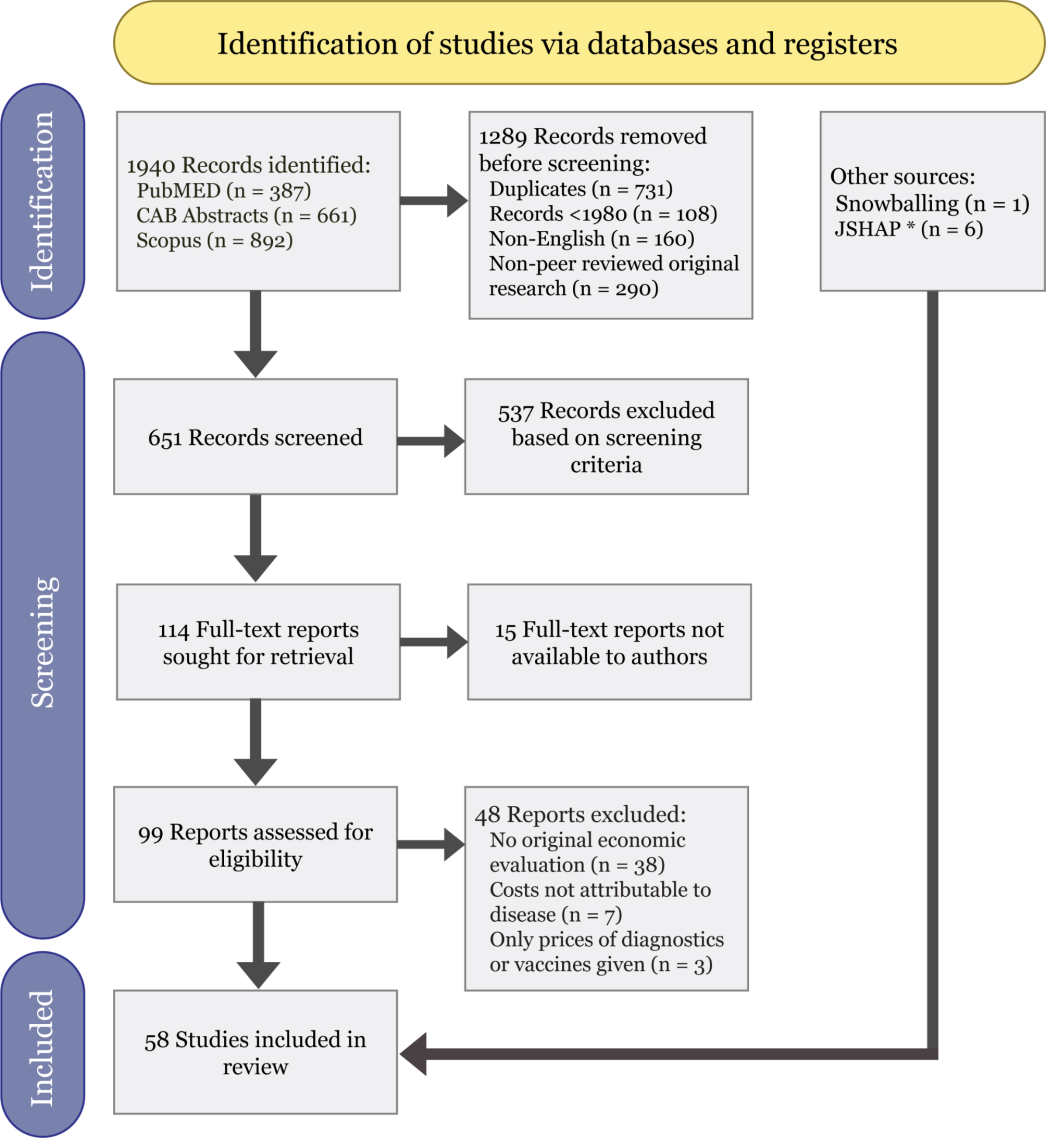
Flow diagram illustrating the systematic search strategy for identifying relevant studies. *JSHAP = Journal of Swine Health and Production

The combination of search terms in the selected databases resulted in 1,940 studies (Fig. 1). In total, 651 non-duplicate citations were screened, and those that did not meet our previously defined screening criteria were excluded, leaving a total of 114 studies. After the final selection, 58 studies were deemed eligible for the purpose of this systematic review, including results from snowballing and JSHAP. The full list of references obtained from the systematic search is available in Supplementary file S2.

### Characteristics of included studies

Detailed characteristics of the studies included in this review are presented in Tables 1 and 2. Overall, the studies were classified into those focused on the economic impact of the disease (23/58; Table 1) and those assessing economics of interventions to prevent and/or control disease (33/58; Table 2). Two studies analysed both the impact of disease and of interventions [20, 21]. Most intervention-focused studies investigated the effects of vaccination (24/35). Of these studies, seventeen evaluated the costs and benefits of vaccination for a short time period (i.e. in one cycle or one year), while seven evaluated the impact for a longer period. After vaccination, the most studied interventions related to elimination strategies (8/35; i.e. depopulation and repopulation, test and removal, herd closure), for all of which the impacts were studied for a long time period (> 1 year). Other interventions that were studied include animal management-related measures (4/35; no mixing of litters, early weaning, selection of pathogen-free gilts, separate housing), medication (3/35), biosecurity (3/35), alternative diet or feed regimen (2/35), and installation of air filtration systems (1/35). Eight of the intervention-focused studies investigated and compared the effects of several interventions.

**Table 1:** Characteristics of studies evaluating the economic impact of disease caused by endemic respiratory pathogens in pigs

Study	Country	Infection scenario	Infectious agent(s)^1^	No. of farms supplying primary production data^2^	Stage of production	Unit of analysis	Study design	Data source(s)^3^	Reference
Pointon et al. (1985)	Australia	Endemic/Epidemic	Mhp	1	Growing	Pig	Controlled trial	DA; C	[26]
Miller and Dorn (1990)	US	Endemic	NS	13	Breeding and growing	Pig	Cross-sectional	DA	[65]
Brouwer et al. (1994)	Netherlands	Endemic	PRRSV	91	Breeding	Sow	Historical control study	DA; C	[66]
Christensen (1995)	New Zealand	Endemic	Mhp	1	Growing	Percentage point of pneumonia	Cross-sectional	DA	[67]
Pejsak and Markowska-Daniel (1997)	Poland	Epidemic	PRRSV	1	Breeding and growing	Farm	Historical control study	DA	[68]
Garner et al. (2001)	Australia	Endemic/Epidemic	PRRSV	NA	Breeding and growing	Country, region, farm, and pig	Stochastic model	LG	[38]
Bennett and IJpelaar (2005)	UK	Endemic	SIV	NA	NR	Country	Deterministic model	LS; E	[69]
Losinger (2005)	US	Endemic/Epidemic	App	NR	Growing	Country	Cobb-douglas production model	DA; LS; LG	[28]
Neumann et al. (2005)	US	Epidemic	PRRSV	10	Breeding and growing	Country and farm	Case-control	DA; LG	[70]
Nieuwenhuis et al. (2012)*	Netherlands	Epidemic	PRRSV	9	Breeding	Sow	Historical control study	DA	[20]
Alarcon, Rushton and Wieland (2013)	UK	Endemic/Epidemic	PCV-2	147	Growing	Country, farm, and pig	Stochastic model	LS; LG; E	[34]
Holtkamp et al. (2013)	US	Endemic	PRRSV	80 breeding farms and 639 groups of growing pigs (No. farms NR)	Breeding and growing	Country, sow, and pig	Cross-sectional	DA; LG	[55]
Nathues et al. (2017)	Germany	Endemic	PRRSV	NA	Breeding and growing	Farm, sow, and pig	Stochastic model	LS; LG; E; C	[35]
Pham et al. (2017) ^4^	Vietnam	Endemic	PRRSV	162	Breeding and growing	Farm	Cross-sectional	DA; E; LS; LG	[22]
Valdes-Donoso et al. (2018)	US	Epidemic	PRRSV	16	Breeding	Farm and sow	Historical control study	DA; LG	[71]
Calderón Díaz, Rodrigues da Costa, et al. (2020)	Ireland	Endemic	NS	56	Growing	Farm, pig, and Kg of meat	Stochastic model	DA; LS	[37]
Calderón Díaz, Fitzgerald, et al. (2020)*	Ireland	Endemic	PRRSV, SIV, Mhp	56	Growing	Farm	Stochastic model	DA	[21]
Ferraz et al. (2020)	Brazil	Endemic	Mhp	1	Growing	Pig	Cross-sectional	DA	[72]
Trevisan et al. (2020)	US	Endemic	PRRSV	20	Breeding and growing	Pig	Cohort	DA; LG	[73]
Paz-Sánchez et al. (2021)	Spain	Endemic	Co-infection	1	Growing	Pig	Cross-sectional	DA; LG	[5]
Renken et al. (2021)	Germany	Endemic	PRRSV	21	Breeding	Farm and sow	Cross-sectional	DA; LS; LG	[39]
Kim et al. (2022)	Korea	Epidemic	PRRSV	1	Breeding and growing	Sow and pig	Historical control study	DA; LS	[74]
Pfuderer et al. (2022)	UK	Endemic	NS	NA	Growing	Pig	Systems dynamics model	DA; LS; LG; A	[27]
Trevisi et al. (2022)	Italy	Endemic	PRRSV	NR	Growing	Pig flow and Kg of meat	Cohort	DA	[75]
Zhang et al. (2022)	China	Epidemic	PRRSV	4	Breeding and growing	Sow and pig	Historical control study	DA; LG	[76]

* Studies focus on both the economic impact of disease and the effects of interventions to prevent/control disease

^1^ PRRSV = porcine reproductive and respiratory syndrome virus; PCV-2 = porcine circovirus 2; SIV = swine influenza virus (SIV); Mhp = *Mycoplasma hyopneumoniae*; App = *Actinobacillus pleuropneumoniae*; NS = not specified

^2^ NR = number of farms providing primary data is not reported; NA = no primary data is provided by farms

^3^ LS = scientific literature; LG = grey literature (e.g. industry reports, websites, proceedings, newsletters, government documents); E = expert(s) opinion; DA = data from authors; C = personal communication; A = author(s) expertise

^4^ Studies consider economic impact in smallholder farm(s) or research facility settings, whereas all other studies consider only commercial farm(s)

**Table 2:** Characteristics of studies evaluating the economic impact of interventions to prevent/control disease caused by endemic respiratory pathogens in pigs

Study	Country	Infection scenario	Infectious agent(s)^1^	Intervention	No. of farms supplying primary production data^2^	Stage of production	Unit of analysis	Study design	Data sources^3^	Reference
Dee (1994)	US	Endemic	Mhp	Medication and early weaning	2	Growing	Pig	Historical control study	DA	[77]
Dee et al. (1996)	US	Endemic	PRRSV	Nursery depopulation	5	Growing	Farm, sow, and pig	Historical control study	DA	[78]
Dee et al. (1997)	US	Endemic	PRRSV	Nursery depopulation	34	Growing	Sow	Historical control study	DA	[79]
Dee and Molitor (1998)	US	Endemic	PRRSV	Elimination (test and removal)	1	Breeding	Sow	Case report	DA; A	[32]
Maes et al. (1998)	Belgium	Endemic	Mhp	Vaccination	5	Growing	Pig	Controlled trial	DA	[42]
Pallares et al. (2000)	Spain	Endemic	Mhp	Vaccination	8	Growing	Pig	Controlled trial	DA	[43]
Kyriakis et al. (2001)	Greece	Endemic	Mhp	Vaccination	1	Growing	Pig	Controlled trial	DA	[80]
Miller et al. (2001)	US	Endemic	Mhp	Vaccination	NA	Growing	Farm	Deterministic model	LS; A	[81]
Pallares et al. (2001)	Spain	Endemic	Mhp	Vaccination	16	Growing	Kg of carcass and kg gained in fattening	Controlled trial	DA	[82]
Maes et al. (2003)	Belgium	Endemic	Mhp	Vaccination	14	Growing	Pig	Controlled trial	DA	[83]
Stipkovits et al. (2003)	Hungary	Endemic	Mhp	Vaccination and medication	1	Growing	Kg of finishing pig marketed	Controlled trial	DA	[84]
Holyoake and Callinan (2006)	Australia	Endemic	Mhp	Vaccination	3	Growing	Pig	Controlled trial	DA; LS; A	[85]
Schaefer and Morrison (2006)	US	Endemic	PRRSV	Elimination (herd closure)	15	Breeding	Herd (farms combined)	Historical control study	DA; A	[86]
Rapp-Gabrielson et al. (2007)	US	Endemic	Co-infection	Vaccination	1	Growing	Carcass value	Controlled trial	DA; LG	[87]
Young et al. (2011) ^4^	Canada	Endemic	PCV-2	Vaccination	1	Growing	Pig	Controlled trial	DA; A	[24]
Nieuwenhuis et al. (2012)*	Netherlands	Epidemic	PRRSV	Monitoring, vaccination, and eradication	9	Breeding	Sow	Historical control study	DA	[20]
Alarcon, Rushton, Nathues, et al. (2013)	UK	Endemic	PCV-2	Vaccination, diets, stocking density, biosecurity, and depopulation-repopulation	50	Growing	Farm	Stochastic model	LS; LG; E	[33]
Alonso et al. (2013)	US	Endemic	PRRSV	Air filtration	21	Breeding	Farm, sow, and pig	Cohort / deterministic model	DA; LS	[30]
Zhang et al. (2014)	Vietnam	Endemic/Epidemic	PRRSV	Vaccination	NA	Growing	Country	Deterministic model	DA; LS; A; E	[57]
Linhares et al. (2015)	US	Endemic	PRRSV	Vaccination	NA	Breeding	Farm	Deterministic & Stochastic model	DA; LS; A	[44]
Ramirez et al. (2015)	US	Endemic	Co-infection	Medication	4	Growing	Pig and Kg of body weight	Controlled trial	DA	[40]
Stygar et al. (2016)	Finland	Epidemic	App	Cleaning, vaccination, and medication	NA	Growing	Pig space unit	Dynamic programming model	LS; A	[88]
Crenshaw et al. (2016)^4^	Canada	Endemic	PRRSV	Feed regimen	1	Growing	Pig	Controlled trial	DA; A	[25]
Kaalberg et al. (2017)	Netherlands	Endemic	Co-infection	Vaccination	1	Growing	Pig	Controlled trial	DA; LG	[89]
Kim et al. (2017)	Korea	Endemic	PRRSV	Vaccination	3	Breeding and growing	Farm	Historical control study	DA; LG	[41]
Zhang et al. (2017)^4^	Cambodia	Epidemic	PRRSV	Vaccination and biosecurity	NA	Breeding and growing	Farm	Deterministic model	DA; LG; LS	[23]
Duivon et al. (2018)	France	Endemic	Co-infection	Vaccination	1	Growing	Sow and pig	Controlled trial	DA; LG	[90]
Nathues et al. (2018)	Germany	Endemic	PRRSV	Depopulation-repopulation, close and roll-over, vaccination, and biosecurity	NA	Breeding and growing	Farm	Stochastic model	LS; A; E	[36]
Silva et al. (2019)	US	Endemic	Mhp	Elimination	70	Breeding and growing	Farm, sow, and pig	Cross-sectional/ deterministic model	DA; LS	[31]
Calderón Díaz, Fitzgerald, et al. (2020)*	Ireland	Endemic	Co-infection	Vaccination	56	Growing	Farm	Stochastic model	DA	[21]
Thomann et al. (2020)	Germany	Endemic	PRRSV	Vaccination	NA	Breeding	Country, farm, and sow	Stochastic model	LS; LG; A	[91]
Abella et al. (2021)	Spain	Endemic/Epidemic	PRRSV	Animal selection	1	Breeding	Sow	Discrete-based event model	DA; LG	[92]
Quezada-Fraide et al. (2021)	Mexico	Endemic	PRRSV	Vaccination	2	Growing	Pig and day of fattening	Cohort study	DA; A	[93]
Jerlström et al. (2022)	Sweden	Endemic	NS	No litter mixing after weaning and separate gilt management	1	Growing	Farm	Deterministic & stochastic model	LG; E, C,	[94]
Moura et al. (2022)	US	Endemic	PRRSV	Vaccination	9	Growing	Experimental group	Controlled trial / deterministic model	DA; LG	[29]

* Studies focus on both the economic impact of disease and the effects of interventions to prevent/control disease

^1^ PRRSV = porcine reproductive and respiratory syndrome virus; PCV-2 = porcine circovirus 2; SIV = swine influenza virus (SIV); Mhp = *Mycoplasma hyopneumoniae*; App = *Actinobacillus pleuropneumoniae*; NS = not specified

^2^ NR = number of farms providing primary data is not reported; NA = no primary data is provided by farms

^3^ LS = scientific literature; LG = grey literature (e.g. industry reports, websites, proceedings, newsletters, government documents); E = expert(s) opinion; DA = data from authors; C = personal communication; A = author(s) expertise

^4^ Studies consider economic impact in smallholder farm(s) or research facility settings, whereas all other studies consider only commercial farm(s)

The studies were conducted in 23 different countries. Twenty-six studies used data derived from European countries, 18 from the US, 6 from Asia, 4 from Oceania, and 4 from other countries, i.e., Canada, Mexico, and Brazil. Considering the period of 1980 until now, we found that over half of the studies (33/58) were published in the last ten years (2013–2022) and, of those, 61% (20/33) focused on PRRSV. Overall, half of the included studies (29/58) analysed the economic impact of PRRSV associated disease and/or its interventions, followed by *M. hyopneumoniae* (13/58). For the remaining pathogens the number of indexed studies was low: three for PCV-2, two for *A. pleuropneumoniae*, and one for SIV. Only in ten of all studies focusing on one specific pathogen, the absence or presence of other specific endemic respiratory pathogens was verified or accounted for. Then, six studies targeted co-infection scenarios (e.g., PRDC). In three of these studies, the co-infection of *M. hyopneumoniae* and PCV-2 was studied, whereas in the remaining studies different combinations of at least three of the primary pathogens (i.e. PRRSV, SIV, PCV-2, *M. hyopneumoniae, A. pleuropneumoniae)* were investigated. Lastly, four studies did not specify the respiratory pathogens involved, instead, they assessed the economic impact of lung lesions. Since the pathogens included in the present review are predominantly endemic worldwide, the economic analyses were mainly applied for endemic scenarios, although 24% (14/58) of the studies also included epidemic (i.e., outbreak) episodes in their analyses.

Most of the studies were conducted in commercial herds (54/58), with only two Asian studies of smallholder farms with less than 20 sows or 100 fattening pigs [22, 23] and two studies conducted in research facilities [24, 25]. The number of farms (owned by one or more pig producers) from which primary data were collected on production performance or health ranged from 1 to 162, with a single farm being investigated in 16 of the studies. Studies on the growing phase (33/58), including weaners and fatteners, predominated over the breeding phase (11/58), although several studies assessed economics in both production phases (14/58). Regarding the expression of economic outcomes, 17 different units of analysis were identified (e.g. pig, herd, farm, Kg meat). In 66% (38/58) of the studies, a singular unit was used, whereas the remaining 34% (20/58) used several units to express economic results. The growing pig was the most extensively used unit of analysis (28/58).

### Methodology applied in included studies

In most of the disease-focused studies (16/25), an observational study design was used in which data were collected cross-sectionally, longitudinally, or retrospectively, with no intervention except for the collection of the data. Of these observational study designs, the cross-sectional study design (7/16) and the historical control study design (6/16), dominated over cohort (2/16) and case-control (1/16) study designs. Across all disease-focused studies, only one controlled trial was performed [26]. The remaining eight studies calculated economic impacts through modelling (8/25); five models were stochastic, one deterministic, one study described the use of a systems dynamics model [27] and one study applied the Cobb-Douglas production function [28]. In three of the modelling studies, input parameters were based on primary data on production performance or health collected on farms. In the remaining five, only secondary data (from scientific literature, grey literature, expert opinion or personal communication) were used. All modelling studies that used secondary data only, performed sensitivity analysis on uncertain input parameters.

Modelling was part of a large share of intervention-focused research, as 11 studies relied on simulation modelling exclusively. Of these studies, four collected primary production data from farms to be used in their models, whereas seven used secondary data only. As before, the modelling studies that used only secondary data performed sensitivity analysis on uncertain input parameters. Additionally, in three intervention-focused studies, controlled trial [29], cohort [30], or cross-sectional [31] study designs were combined with an economic deterministic model. Furthermore, fourteen studies collected data solely by means of a controlled trial and six by means of observational study designs (five historical control studies and one cohort study). One study, by Dee and Molitor [32], entailed a case report describing the attempt of PRRSV elimination on one farm. For detailed information on the study designs per included study, refer to Tables 1 and 2.

Economic methods that were applied in the eligible studies, ranged from basic cost computations to more comprehensive methods commonly used in animal health economics (Table 3). The most utilised methods were basic cost computations (15/58) and cost analysis (14/58), followed by partial budget analysis (12/58). As expected, methods built for comparing the profitability of on-farm changes, i.e. the partial budget and cost-benefit analysis, were almost exclusively applied in intervention-focused studies. In five modelling studies, multiple economic methods were applied [33–37].

**Table 3:** Economic evaluation methods used in the eligible studies

Method	Description	Number of studies (D|I)*
Basic computation	Basic calculation of a cost (e.g., total vaccination purchase costs) or of a reduction in revenues (e.g., reduction of number of piglets weaned * sale price per piglet); or adjustment of one value in an external tool.	5 | 10
Cost analysis	Calculation or estimation of several or total variable costs (including estimation of reduced revenues).	9 | 5
Margin over specific variable costs	Revenue minus feed and/or veterinary (medication/vaccination) costs.	0 | 5
Gross margin	Enterprise outputs minus all variable costs^1^.	5 | 4
Farm budget	Calculation of the total net profit on a farm, by deducting fixed costs from the gross margin^1^.	5 | 2
Partial budget	Determining the change in net profit resulting from a change on a farm. Calculated by identifying revenues foregone, extra costs, additional revenue, and reduced costs^1^.	2 | 10
Cost-benefit analysis	Determining the profitability of programs over an extended period of time, by enumerating benefits and costs and applying a discount rate to convert future values to present values. Consequently, the Net Present Value and Benefit-Cost Ratio can be calculated^1^.	0 | 2
Other	Types of economic analysis performed by single studies. 1. Economic welfare analysis 2. Cash flow analysis and decision optimization.	1. Disease-focused. (28) 2. Intervention- focused. (33)

* D = Disease-focused, I = Intervention-focused

^1^ Description derived from Dijkhuizen, Morris and Huirne (95)

Seven studies provided estimates of the economic burden at a national level, of which only two studies included price effects across the industry or looked at changes in consumer and producer surplus due to decreased pork production [28, 38]. The remaining five studies extrapolated farm-level estimates without accounting for additional macro-economic effects. Thus, most studies investigated the financial losses at the farm-level, rather than economic losses. However, to keep the terminology simple, we will keep referring to the calculated impacts as the economic impact.

**Fig. 2:**
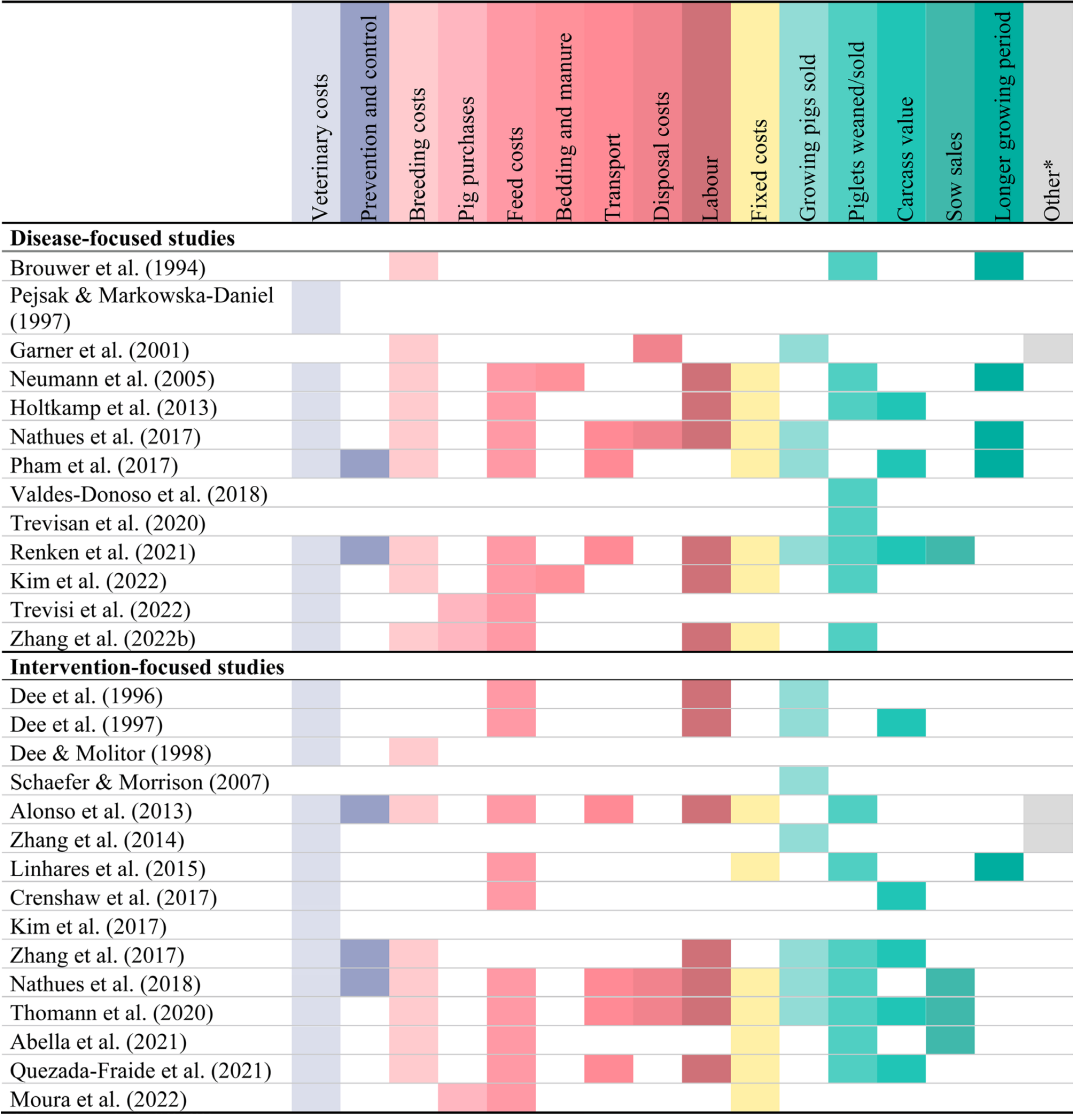
Cost and revenue components considered in economic analyses of studies on PRRSV. * Other components include penalties, subsidies/compensation and industry effects

To calculate the on-farm economic impact, a wide range of cost components were considered across all papers (for detailed information of the components per study, please refer to Supplementary file S3). Studies using the same economic method or focusing on the same disease, often included different cost and revenue components in their calculations (Fig. 2). Overall, the most frequently used cost components were veterinary costs (49/58 studies), feed costs (39/58), and labour costs (26/58); whereas the most frequently used revenue components were reduced carcass value (24/58), fewer growing pigs sold (19/58) and fewer piglets weaned/sold (19/58). The modelling studies that considered the most cost components [33, 36, 37, 39] all reported that feed costs and the reduced revenue from fewer sold piglets or fattening pigs were the costliest components. Although most studies included these components, 19 out of the 58 studies did not consider feed costs, and 24 did not calculate lost revenues due to fewer piglets weaned or fattening pigs sold.

### Pathogen specific costs

**Fig. 3:**
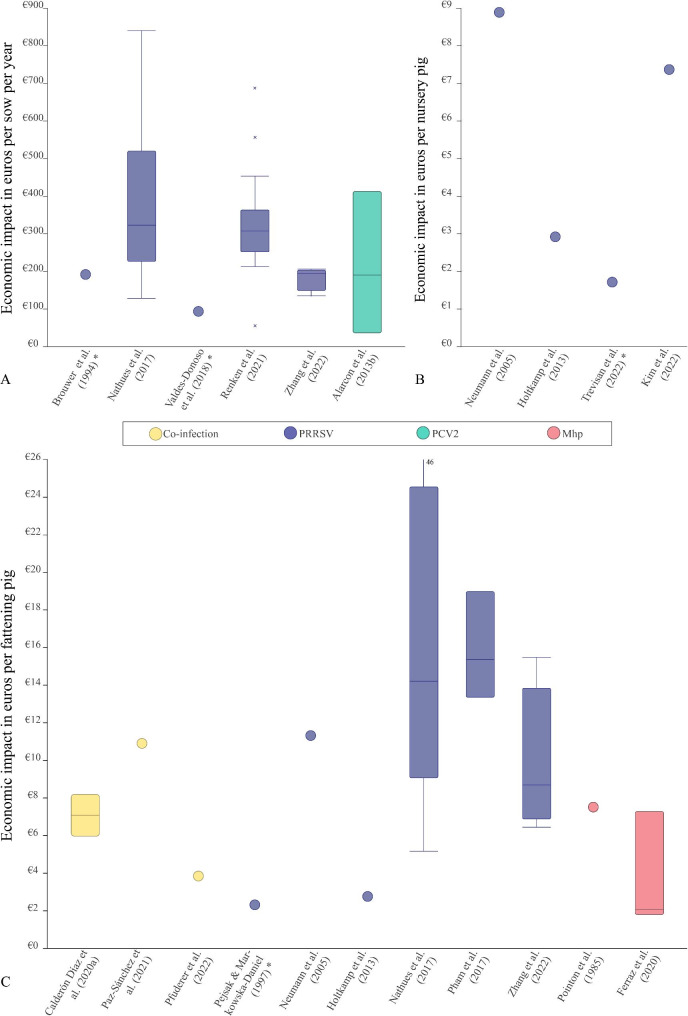
Economic impact of disease caused by endemic respiratory pathogens. The economic impact is expressed in decreased profit (in euros) per sow-year (**a**), per nursery pig (**b**), and/or, per fattening pig (**c**). Circles indicate a single reported outcome, whereas boxplots represent a range of economic outcomes from one study (e.g. when different scenarios with varying disease severity were considered, or when economic losses were reported for multiple farms separately). Reported outcomes were adjusted for inflation up until the year 2023 and converted to euros as a common currency. Studies that are marked with an *, did not include feed costs as a component in their economic analysis

Despite the variety in units of analysis, the economic outcomes per study could be converted to a common unit for 17 out of 25 disease-focused studies (Fig. 3a-c). This figure serves as an illustration for the range in reported economic impacts, but it should be noted that study outcomes cannot be merged directly due to the variety in study characteristics and methods of calculation.

Since most intervention-focused studies analysed the benefits of vaccination, the main economic outcomes for these studies are summarised in Table 4. It is evident from this table that there is no common method for expressing the main economic impact of vaccination. Overall, most of the intervention-focused studies (24/35) reported a positive economic impact due to the implementation of the respective intervention, while three reported a negative impact [21, 32, 40] and four a neutral impact [30, 41–43]. In the remaining four intervention-focused studies, the effects of different interventions were compared with each other rather than with a control group [20, 25, 29, 44]. For all outcomes from both disease-focused and intervention-focused studies in their original currency, please refer to Supplementary file S4.

**Table 4:** Economic impact of vaccination against endemic respiratory diseases in pigs

Study	Economic outcome in euros	Unit		Reference
**Co-infections**				
Rapp-Gabrielson et al. (2007)^1^	12.91, 7.82, 9.57	Increased value per carcass for three different vaccines (compared to control)		[87]
Kaalberg et al. (2017)^2^	3.67	Benefit per finisher		[89]
Duivon et al. (2018)^2^	2.16	Benefit per finisher		[90]
**Porcine reproductive and respiratory syndrome virus**				
Zhang et al. (2014)	2.3–4.5	Benefit-cost ratio		[57]
Moura et al. (2022)	1.83	Benefit-cost ratio		[29]
Kim et al. (2017)		Difference in medication costs not significant		[41]
Linhares et al. (2015)	32,345	Difference in opportunity costs between modified-live virus vaccination and field-virus inoculation for a 1,000 sow breeding herd		[44]
Zhang et al. (2017)	155.20-316.68	Increased net profits per farm (two-sow breeder; five-pig fattener; single-sow, three-pig farrow-to-finish)		[23]
Thomann et al. (2020)	1) 211–422 2) 184–335	Median annual benefits per sow of (1) vaccinating sows and piglets and (2) Vaccinating only sows		[91]
Quezada-Fraide et al. (2021)	2.14	Difference in costs per weaned pig between vaccinating sows and piglets and vaccinating sows only		[93]
***Mycoplasma hyopneumoniae***				
Maes et al. (1998)		Difference in curative parental medication costs not significant		[42]
Pallarés et al. (2000)		Difference in medication costs not significant		[43]
Kyriakis et al. (2001)	0.46, 0.36	Reduced medication cost per pig for two different vaccination schemes (compared to control)		[80]
Stipkovits et al. (2003)	1) -0.02, -0.06 2) 0.03, 0.08	Difference in margin over feed and medication costs per kg of finishing pig marketed for vaccinating (1) once or (2) twice (compared to 2 control groups)		[84]
Maes et al. (2003)	1.17	Additional return to labour per pig		[83]
Holyoake and Callinan (2006)	4.91	Increase in profit per pig		[85]
Miller et al. (2001)	1) 4,978 2) 13,056	Increased annual profits for farms (1020 fatteners placed) shipping (1) by target weight or (2) on fixed date		[81]
**Porcine circovirus 2**				
Young et al. (2011)	7.57	Return on investment from vaccination per pig		[24]
Alarcon, Rushton, Nathues, et al. (2013)	1) 24,853 2) 97,206	Mean expected value of vaccination after 5 years for a (1) moderately affected farm (100 sows), (2) severely affected farm (100 sows)		[33]

Reported outcomes were adjusted for inflation up until the year 2023 and converted to euros as a common currency

^1^ Pigs were vaccinated against *Mycoplasma hyopneumoniae*

^2^ Pigs were vaccinated against *Mycoplasma hyopneumoniae* and porcine circovirus type 2

## Discussion

An economic perspective is key to understand the impacts of disease and the intervention options available, and, therefore, to improve decision-making regarding animal health and welfare. This is especially important when endemic diseases are concerned, since their effects are often not easily quantified [45]. The present systematic review is the first in the field aiming to identify the economic impacts of specific or co-existing pathogens involved in the porcine respiratory disease complex (PRDC), and the costs and benefits of interventions. This work additionally reveals the economic evaluation methods that were applied across included studies, including the cost and revenue components that were considered in their calculations.

In an ideal scenario, an estimated disease impact should be completely attributable to the disease that is being analysed. However, often endemic respiratory diseases are multifactorial, and the impact of the disease can be influenced by multiple non-infectious risk factors. In addition, pig herds are often burdened with more than one endemic respiratory disease at the same time under the umbrella of the PRDC [12, 13]. If the whole complex is not carefully studied, this could result in flawed estimates. Consequently, studying the effects of a specific pathogen where multiple disease-causing factors are involved is rather difficult, if not impossible in many cases. Most studies in the present review focused on one respiratory pathogen, and the presence or absence of other pathogen(s) was often not established. Therefore, the reported economic outcomes may not fully be the result of one specific respiratory pathogen only, but will be the product of a complex interaction between infectious agents, management conditions, environment, and genetics [12, 13].

In total, 58 peer-reviewed studies were included within this systematic review. Most of these studies analysed the effects of an intervention, of which nearly half focused on vaccination. With fairly low numbers of studies on PCV-2, *A. pleuropneumoniae* and SIV, the PRRSV was by far the most studied pathogen, followed by *M. hyopneumoniae*. However, it should be noted that most studies on PRRSV were from the United States, thus outcomes were based on the effects of PRRSV-2 genotypes, which tend to be considered more virulent than PRRSV-1 ones, predominantly present in Europe [46]. However, others could not confirm that PRRSV-2 genotypes are more virulent than PRRSV-1 [47, 48]. Nevertheless, estimates of PRRSV impact might be overestimated due to the overrepresentation of studies based on PRRSV-2. Although the difference in strain virulence of PRRSV-1 and PRRSV-2 genotypes shows perhaps the most clear difference in disease impact due to differentiated virus species, many studies have shown a variety of genotypes for a respiratory pathogen circulating and evolving within continents, countries, and even within the same swine operation over time [49–52]. The evolution of genotypes may influence not only their virulence, but also their resistance to treatments and vaccine efficacy [53, 54].

An additional factor adding to the variation in economic impact is the variety in production systems and the overall industry structure across countries. Comparing the production losses on a commercial fattening farm in the United States [55] to the losses for a smallholder breeding farm in Vietnam [22] provides an evident example, but even within a continent or country vast differences may exist due to, among others, varying genetics of the pigs (e.g. differing productivity or disease resilience), the internal and external climate, the farm’s biosecurity or health status, access to high quality raw materials and veterinary services, differing target weights for selling and the size of the farm. External factors such as the amount of international import and export and governmental subsidies or other incentives can also lead to differences in economic losses suffered by the industry due to endemic respiratory disease. As this review covers research from a period of nearly 40 years, the evolution of pig production systems and industries regarding these aspects should be considered when drawing conclusions. It should be stressed that, although the described variation may complicate comparing or merging of study outcomes by means of a meta-analysis [56], this variation in research is essential to understand the range in economic impact from endemic respiratory disease at a global level.

When translating production impact into financial consequences, various limitations arise regarding the applied economic methodology. We observed over seven different economic evaluation methods with a large variety in cost and revenue components used to calculate economic outcomes. With the exception of one study [57], in which the farmers’ willingness to pay for a vaccine was estimated, the studies included in this review did not include non-monetary costs (e.g. environmental, social or welfare effects). The methods applied in the eligible studies varied from basic cost calculations to more comprehensive methods such as a farm budget analysis. Even after grouping eligible studies by their applied economic method, it was rare that the same cost and revenue components were used. Although we assume that for most studies, the authors included the components that were most relevant for the specific farms under study, a highly varying level of detail in calculations impacts the comparability of economic outcomes from each study. For instance, while increased feed costs and reduced revenue from fewer weaned or sold pigs were identified as the most important components [33, 36, 37, 39], over a third of all studies did not include one or both components. Although these studies do not provide a specific reason for not including these components, it is recognised that in a number of them calculating the economic impact of a disease or an intervention was not the primary objective. Leaving out these important cost components may, therefore, be suitable for their respective study aims, but referring to the results as true economic impact estimates will lead to biased conclusions and comparisons with other study outcomes, as the total costs are underestimated. Additionally, the amount of feed costs per kg of carcass can differ greatly between countries, especially between continents [58]. This fact additionally holds for revenues per kg of carcass and the costs of medicines and vaccines [58, 59]. Moreover, the prices of feed and raw materials are volatile and particularly rising in Europe during the last few years [60], which further impacts the comparability of economic outcomes estimated during different time periods.

While keeping the differences in economic evaluation methods, their level of detail and the differences in prices across countries and time in mind, most outcomes from the disease-focused studies could be converted to an economic impact in euros per pig, which gives a very rough impression of the range in economic impact of the PRDC syndrome. The median economic impact of one or several co-existing respiratory pathogen(s) as extracted from all studies, ranged from €1.70 to €8.90 per nursery pig, €2.30 to €15.35 per fattening pig, and €100 to €323 per sow per year. Excluding the studies in which feed costs were not considered, increases the minimum reported costs to €2.90 per nursery pig, €2.80 per fattening pig, and €195 per sow per year. Due to the low numbers of studies on pathogens other than PRRSV, the ranges mainly reflect the significant worldwide impact of PRRSV. It is, therefore, unfeasible to compare and rank the various pathogens according to their economic importance. Furthermore, converting absolute economic outcomes to a single currency complicates the interpretability and comparability of the study outcomes, as differences exist in the relative importance of the economic losses suffered by farmers from countries of different income levels. Preferably, outcomes would be reported in a relative manner, such as the percent decrease in profits due to disease. However, most often information on farm profits in a non-diseased scenario is lacking.

Nearly all studies reported neutral or positive impacts of implementing an intervention. This suggests that for a wide range of production systems and disease scenarios, implementing an intervention on a farm with endemic respiratory diseases increases farm profits. There may be an outcome reporting bias, with only the favourable interventions reported that can undermine the validity of systematic reviews [61]. However, we have no evidence that this is the case in our systematic review. Apparently, most studies looked at the effects of vaccination, with very few studies considering long-term sustainable interventions. Where several countries are making efforts to eliminate endemic respiratory diseases completely [62, 63], economic research on long-term interventions (such as improvement of management practices, housing conditions or biosecurity measures) would provide valuable information for countries starting with or expanding the elimination of endemic respiratory pathogens. Besides the low number of studies on an intervention other than vaccination, comparison and ranking of interventions was also made unfeasible by the variation in the expression of results. Future research should use more standardised approaches for economic analyses of interventions with similar outcome metrics. For instance, in human health economics, comparison of control programs is mainly done by determining the cost-effectiveness (e.g. costs per disability-adjusted life-year) or cost-benefit ratio [59]. In the case of interventions requiring a large initial investment, calculations of the payback period or return on investment might be preferred [59].

Although the benefits from a standardised approach seem clear from discussing the limitations in the existing research, developing such an approach poses a challenge. The choice for a specific economic method is often dependent on the data available for the study, as well as the purpose of the study outcomes and the nature of the decision (whether researchers estimate the economic impact at the micro-scale or macro-scale, and for a short- or long-term, etc.). Consequently, the richness in methods could be an advantage, rather than only a limitation, as it will allow better alignment of the studies to the decision process required. It would therefore be of great interest to investigate why different methods or outcomes were chosen over others. Moreover, the industry-level economic burden of respiratory diseases in pigs is not limited to the direct costs, but also includes indirect costs, such as costs suffered by non-affected farms due to biosecurity investments or fluctuations in availability and prices of inputs and outputs. Most studies included in this systematic review focused on farm-level economic impacts, whereas methods well suited to study industry effects, such as the partial equilibrium analysis and econometric models, have not yet been explored. Likewise, economic analyses of the impact of policies to control PRDC pathogens were not found through the search. Therefore, there is currently no clarity on which indirect cost and revenue components from the PRDC seem to be most impactful at industry level. An approach that enhances the understanding of the economic burden of endemic respiratory disease for the entire industry would ideally include a range of economic methods, that captures both the economic impact on the farm, and on the (national or international) industry. Such an approach is being taken by the Global Burden of Animal Diseases programme and is being tested in different parts of the world [62, 63].

Lastly, restricting the review to only peer-reviewed English literature ensures a certain quality of the work but can also narrow the scope of the review and the results. Including “grey” literature during the search, such as conference abstracts and industry reports, would mostly provide additional cost estimations by non-academic organisations or companies. This could assist with reducing publication bias, but it is important to ensure that the study is relevant to the research question and that it is of sufficient quality to be included in the review [64]. In this case, several non-peer-reviewed sources were identified, but oftentimes these entailed works in progress, pilot studies, or works that did not contain adequate or complete information (e.g. explicit information on cost or revenue components). This, together with the fact that searching for abstracts is resource-intensive and availability is usually compromised, advocated for the inclusion of peer-reviewed records only.

In conclusion, respiratory diseases represent a significant economic burden in pig production, as highlighted by the range in economic impact provided in this systematic review. Future research should improve the consistency and comparability of economic assessments by ensuring the inclusion of high impact cost and revenue components and expressing results similarly. Regardless, the outcomes from this systematic review provide insight in the variation in studies, their methods, their advantages and limitations, and the reported impacts from the endemic respiratory disease complex for pig production systems worldwide.

### Electronic supplementary material

Below is the link to the electronic supplementary material.

**Supplementary file S1.** Terms used to build the full search strings. - File provides a table of the terms that were used in the search for eligible literature

**Supplementary file S2.** List of eligible studies. - File provides the full list of studies included in the systematic review

**Supplementary file S3.** Economic methods and cost components per study. - File provides full details on which economic method was applied and which cost components were considered per study

**Supplementary file S4.** Reported economic outcomes per study. - File provides the economic outcomes in their original valuta as reported in each disease-focused and intervention-focused study. The file additionally includes information on the evaluation period for intervention-focused studies and on whether the economic analysis accounts for the presence/absence of other PRDC pathogens

## Data Availability

Data sharing is not applicable to this article as no datasets were generated or analysed during the current study. All created tables supporting the conclusions of this article are included within the article and its supplementary files.

## Abbreviations

A. pleuropneumoniae: Actinobacillus pleuropneumoniae
JSHAP: Journal of Swine Health and Production
M. hyopneumoniae: Mycoplasma hyopneumoniae
PCV-2: Porcine circovirus 2
PRDC: Porcine respiratory disease complex
PRRSV: Porcine reproductive and respiratory syndrome virus
SIV: Swine influenza virus
