# Supplementary material for: The economic impact of endemic respiratory disease in pigs and related interventions - a systematic review
Source: Porcine Health Manag. 2023 Oct 17;9:45. doi: 10.1186/s40813-023-00342-w (PMC10583309; doi:10.1186/s40813-023-00342-w)
Supplement: Supplementary file 1 — Supplementary file S1. Terms used to build the full search strings. - File provides a table of the terms that were used in the search for eligible literature [file 40813_2023_342_MOESM1_ESM.docx]

**Supplementary File S1. Terms used to build the full search strings.**

| **Topic** | **Population** | **Focus** |
| --- | --- | --- |
| “Respiratory disease” OR pneumonia  OR  “Porcine Reproductive and Respiratory Syndrome” OR PRRSV  OR  “Porcine Circovirus” OR PCV2 OR PCVAD OR“Postweaning Multisystemic Wasting Syndrome”  OR  “Swine Influenza” OR SIV OR “Swine flu” OR “Pig Influenza”  OR  *“Mycoplasma hyopneumoniae”* OR Mhyo  OR  *“Actinobacillus pleuropneumoniae”* | Swine OR pig* | Economic* OR cost OR costs OR financial OR finance* OR cost-benefit OR benefit-cost OR profit OR benefit OR margin |
