# Supplementary material for: The economic impact of endemic respiratory disease in pigs and related interventions - a systematic review
Source: Porcine Health Manag. 2023 Oct 17;9:45. doi: 10.1186/s40813-023-00342-w (PMC10583309; doi:10.1186/s40813-023-00342-w)
Supplement: Supplementary file 2 — Supplementary file S2. List of eligible studies. - File provides the full list of studies included in the systematic review [file 40813_2023_342_MOESM2_ESM.docx]

**Supplementary File S2 – List of eligible studies**

1. Abella, G., Pagès-Bernaus, A., Estany, J., Pena, R. N., Fraile, L., & Plà-Aragonés, L. M. (2021). Using PRRSV-resilient sows improve performance in endemic infected farms with recurrent outbreaks. *Animals*, *11*(3), 1–16. https://doi.org/10.3390/ani11030740
2. Alarcon, P., Rushton, J., Nathues, H., & Wieland, B. (2013). Economic efficiency analysis of different strategies to control post-weaning multi-systemic wasting syndrome and porcine circovirus type 2 subclinical infection in 3-weekly batch system farms. *Preventive Veterinary Medicine*, *110*(2), 103–118. https://doi.org/10.1016/j.prevetmed.2012.12.006
3. Alarcon, P., Rushton, J., & Wieland, B. (2013). Cost of post-weaning multi-systemic wasting syndrome and porcine circovirus type-2 subclinical infection in England – An economic disease model. *Preventive Veterinary Medicine*, *110*(2), 88–102. https://doi.org/10.1016/j.prevetmed.2013.02.010
4. Alonso, C., Davies, P. R., Polson, D. D., Dee, S. A., & Lazarus, W. F. (2013). Financial implications of installing air filtration systems to prevent PRRSV infection in large sow herds. *Preventive Veterinary Medicine*, *111*(3–4), 268–277. https://doi.org/10.1016/j.prevetmed.2013.05.001
5. Bennett, R., & Ijpelaar, J. (2005). Updated estimates of the costs associated with thirty-four endemic livestock diseases in Great Britain: A note. *Journal of Agricultural Economics*, *56*(1), 135–144. https://doi.org/10.1111/j.1477-9552.2005.tb00126.x
6. Brouwer, J., Frankena, K., de Jong, M. F., Voets, R., Dijkhuizen, A., Verheijden, J., & Komijn, R. E. (1994). PRRS: Effect on herd performance after initial infection and risk analysis. *Veterinary Quarterly*, *16*(2), 95–100. https://doi.org/10.1080/01652176.1994.9694427
7. Calderón Díaz, J. A., Fitzgerald, R. M., Shalloo, L., Rodrigues da Costa, M., Niemi, J., Leonard, F. C., Kyriazakis, I., & García Manzanilla, E. (2020). Financial analysis of herd status and vaccination practices for porcine reproductive and respiratory syndrome virus, swine influenza virus, and *Mycoplasma hyopneumoniae* in farrow-to-finish pig farms using a bio-economic simulation model. *Frontiers in Veterinary Science*, *7*, 556674. https://doi.org/10.3389/fvets.2020.556674
8. Calderón Díaz, J. A., Rodrigues da Costa, M., Shalloo, L., Niemi, J. K., Leonard, F. C., Crespo-Piazuelo, D., Gasa, J., & García Manzanilla, E. (2020). A bio-economic simulation study on the association between key performance indicators and pluck lesions in Irish farrow-to-finish pig farms. *Porcine Health Management*, *6*(1), 40. https://doi.org/10.1186/s40813-020-00176-w
9. Christensen, N. H. (1995). Evaluation of the effects of enzootic pneumonia in pigs on weight gain and days to slaughter under New Zealand conditions. *New Zealand Veterinary Journal*, *43*(4), 146–148. https://doi.org/10.1080/00480169.1995.35875
10. Crenshaw, J., Campbell, J., Polo, J., & Bussières, D. (2017). Effects of a nursery feed regimen with spray-dried bovine plasma on performance and mortality of weaned pigs positive for porcine reproductive and respiratory syndrome virus. *Journal of Swine Health and Production*, *25*(1), 10–18.
11. Dee, S. (1994). Apparent prevention of *Mycoplasma hyopneumoniae* infection in growing pigs with a low-cost modified medicated-early-weaning program. *Journal of Swine Health and Production*, *2*(6), 7–12.
12. Dee, S. A., Joo, H. S., & Polson, D. D. (1996). Improved performance of a large pig complex after sequential nursery depopulation. *Veterinary Record*, *138*(2), 31–34. https://doi.org/10.1136/vr.138.2.31
13. Dee, S. A., Joo, H. S., Polson, D. D., & Marsh, W. E. (1997). Evaluation of the effects of nursery depopulation on the profitability of 34 pig farms. *Veterinary Record*, *140*(19), 498–500. https://doi.org/10.1136/vr.140.19.498
14. Dee, S. A., & Molitor, T. W. (1998). Elimination of porcine reproductive and respiratory syndrome virus using a test and removal process. *Veterinary Record*, *143*(17), 474–476. https://doi.org/10.1136/vr.143.17.474
15. Duivon, D., Corrégé, I., Hémonic, A., Rigaut, M., Roudaut, D., & Jolie, R. (2018). Field evaluation of piglet vaccination with a *Mycoplasma hyopneumoniae* bacterin as compared to a ready-to-use product including porcine circovirus 2 and *M. hyopneumoniae* in a conventional French farrow-to-finish farm. *Porcine Health Management*, *4*(4), (18 January 2018). https://doi.org/10.1186/s40813-017-0077-y
16. Ferraz, M. E. S., Almeida, H. M. S., Storino, G. Y., Sonálio, K., Souza, M. R., Moura, C. A. A., Costa, W. M. T., Lunardi, L., Linhares, D. C. L., & de Oliveira, L. G. (2020). Lung consolidation caused by *Mycoplasma hyopneumoniae* has a negative effect on productive performance and economic revenue in finishing pigs. *Preventive Veterinary Medicine*, *182*, 105091. https://doi.org/10.1016/j.prevetmed.2020.105091
17. Garner, M. G., Whan, I. F., Gard, G. P., & Phillips, D. (2001). The expected economic impact of selected exotic diseases on the pig industry of Australia. *Revue Scientifique et Technique - Office International Des Épizooties*, *20*(3), 671–685. https://doi.org/10.20506/rst.20.3.1303
18. Holtkamp, D. J., Kliebenstein, J. B., Neumann, E. J., Zimmerman, J. J., Rotto, H. F., Yoder, T. K., Wang, C., Yeske, P. E., Mowrer, C. L., & Haley, C. A. (2013). Assessment of the economic impact of porcine reproductive and respiratory syndrome virus on United States pork producers. *Journal of Swine Health and Production*, *21*(2), 72–84.
19. Holyoake, P. K., & Callinan, A. P. L. (2006). How effective is *Mycoplasma hyopneumoniae* vaccination in pigs less than three weeks of age? *Journal of Swine Health and Production*, *14*(4), 189–195.
20. Jerlström, J., Huang, W., Ehlorsson, C.-J., Eriksson, I., Reneby, A., & Comin, A. (2022). Stochastic partial budget analysis of strategies to reduce the prevalence of lung lesions in finishing pigs at slaughter. *Frontiers in Veterinary Science*, *9*. https://doi.org/10.3389/fvets.2022.957975
21. Kaalberg, L., Geurts, V., & Jolie, R. (2017). A field efficacy and safety trial in the Netherlands in pigs vaccinated at 3 weeks of age with a ready-to-use porcine circovirus type 2 and *Mycoplasma hyopneumoniae* combined vaccine. *Porcine Health Management*, *3*(1), 23. https://doi.org/10.1186/s40813-017-0070-5
22. Kim, J. H., Kim, S. C., Kim, H. J., Jeong, C. G., Park, G. S., Choi, J. S., & Kim, W. I. (2022). Insight into the economic effects of a severe Korean PRRSV1 outbreak in a farrow-to-nursery farm. *Animals*, *12*(21). https://doi.org/10.3390/ani12213024
23. Kim, J., Lee, J.-A., Choi, H., Han, J., Huh, W., Pi, J.-H., Lee, J.-K., Park, S., Cho, K., & Lee, J. (2017). *In vitro* and *in vivo* studies of deglycosylated chimeric porcine reproductive and respiratory syndrome virus as a vaccine candidate and its realistic revenue impact at commercial pig production level. *Vaccine*, *35*(37), 4966–4973. https://doi.org/10.1016/j.vaccine.2017.07.075
24. Kyriakis, S. C., Alexopoulos, C., Vlemmas, J., Sarris, K., Lekkas, S., Koutsoviti-Papadopoulou, M., & Saoulidis, K. (2001). Field study on the efficacy of two different vaccination schedules with HYORESP in a *Mycoplasma hyopneumoniae*-infected commercial pig unit. *Journal of Veterinary Medicine Series B*, *48*(9), 675–684. https://doi.org/10.1046/j.1439-0450.2001.00494.x
25. Linhares, D. C., Johnson, C., & Morrison, R. B. (2015). Economic analysis of immunization strategies for PRRS control [corrected]. *PLoS ONE*, *10*(12), e0144265. https://doi.org/10.1371/journal.pone.0144265
26. Losinger, W. C. (2005). Economic impacts of reduced pork production associated with the diagnosis of *Actinobacillus pleuropneumoniae* on grower/finisher swine operations in the United States. *Preventive Veterinary Medicine*, *68*(2/4), 181–193. https://doi.org/10.1016/j.prevetmed.2004.12.004
27. Maes, D., Deluyker, H., Verdonck, M., Castryck, F., Miry, C., Lein, A., Vrijens, B., & Kruif, A. de. (1998). The effect of vaccination against *Mycoplasma hyopneumoniae* in pig herds with a continuous production system. *Journal of Veterinary Medicine. Series B*, *45*(8), 495–505. https://doi.org/10.1111/j.1439-0450.1998.tb00820.x
28. Maes, D., Verbeke, W., Vicca, J., Verdonck, M., & de Kruif, A. (2003). Benefit to cost of vaccination against *Mycoplasma hyopneumoniae* in pig herds under Belgian market conditions from 1996 to 2000. *Livestock Production Science*, *83*(1), 85–93. https://doi.org/10.1016/S0301-6226(03)00039-3
29. Miller, G. Y., & Dorn, C. R. (1990). Costs of swine diseases to producers in Ohio. *Preventive Veterinary Medicine*, *8*(2–3), 183–190. https://doi.org/10.1016/0167-5877(90)90010-F
30. Miller, G. Y., Song, Y., & Bahnson, P. B. (2001). An economic model for estimating batch finishing system profitability with an application in estimating the impact of preventive measures for porcine respiratory disease complex. *Journal of Swine Health and Production*, *9*(4), 169–177.
31. Moura, C. A. A., Philips, R., Silva, G. S., Holtkamp, D. J., & Linhares, D. C. L. (2022). Comparison of virus detection, productivity, and economic performance between lots of growing pigs vaccinated with two doses or one dose of PRRS MLV vaccine, under field conditions. *Preventive Veterinary Medicine*, *204*, 105669. https://doi.org/10.1016/j.prevetmed.2022.105669
32. Nathues, H., Alarcon, P., Rushton, J., Jolie, R., Fiebig, K., Jimenez, M., Geurts, V., & Nathues, C. (2017). Cost of porcine reproductive and respiratory syndrome virus at individual farm level – An economic disease model. *Preventive Veterinary Medicine*, *142*, 16–29. https://doi.org/10.1016/j.prevetmed.2017.04.006
33. Nathues, H., Alarcon, P., Rushton, J., Jolie, R., Fiebig, K., Jimenez, M., Geurts, V., & Nathues, C. (2018). Modelling the economic efficiency of using different strategies to control porcine reproductive & respiratory syndrome at herd level. *Preventive Veterinary Medicine*, *152*, 89–102. https://doi.org/10.1016/j.prevetmed.2018.02.005
34. Neumann, E. J., Kliebenstein, J. B., Johnson, C. D., Mabry, J. W., Bush, E. J., Seitzinger, A. H., Green, A. L., & Zimmerman, J. J. (2005). Assessment of the economic impact of porcine reproductive and respiratory syndrome on swine production in the United States. *Journal of the American Veterinary Medical Association*, *227*(3), 385–392. https://doi.org/10.2460/javma.2005.227.385
35. Nieuwenhuis, N., Duinhof, T. F., & Nes, A. van. (2012). Economic analysis of outbreaks of porcine reproductive and respiratory syndrome virus in nine sow herds. *Veterinary Record*, *170*(9), 225. https://doi.org/10.1136/vr.100101
36. Pallarés, F. J., Gómez, S., & Muñoz, A. (2001). Evaluation of the zootechnical parameters of vaccinating against swine enzootic pneumonia under field conditions. *Veterinary Record*, *148*(4), 104–107. https://doi.org/10.1136/vr.148.4.104
37. Pallarés, F. J., Gómez, S., Ramis, G., Seva, J., & Muñoz, A. (2000). Vaccination against swine enzootic pneumonia in field conditions: Effect on clinical, pathological, zootechnical and economic parameters. *Veterinary Research*, *31*(6), 573–582. https://doi.org/10.1051/vetres:2000141
38. Paz-Sánchez, Y., Herráez, P., Quesada-Canales, Ó., Poveda, C. G., Díaz-Delgado, J., Quintana-Montesdeoca, M. del P., Plamenova Stefanova, E., & Andrada, M. (2021). Assessment of lung disease in finishing pigs at slaughter: Pulmonary lesions and implications on productivity parameters. *Animals*, *11*(12), 3604. https://doi.org/10.3390/ani11123604
39. Pejsak, Z., & Markowska-Daniel, I. (1997). Losses due to porcine peproductive and respiratory syndrome in a large swine farm. *Comparative Immunology, Microbiology and Infectious Diseases*, *20*(4), 345–352. https://doi.org/10.1016/S0147-9571(97)00010-6
40. Pfuderer, S., Bennett, R. M., Brown, A., & Collins, L. M. (2022). A flexible tool for the assessment of the economic cost of pig disease in growers and finishers at farm level. *Preventive Veterinary Medicine*, *208*, 105757. https://doi.org/10.1016/j.prevetmed.2022.105757
41. Pham, H. T. T., Antoine-Moussiaux, N., Grosbois, V., Moula, N., Truong, B. D., Phan, T. D., Vu, T. D., Trinh, T. Q., Vu, C. C., Rukkwamsuk, T., & Peyre, M. (2017). Financial impacts of priority swine diseases to pig farmers in Red river and Mekong river delta, Vietnam. *Transboundary and Emerging Diseases*, *64*(4), 1168–1177. https://doi.org/10.1111/tbed.12482
42. Pointon, A. M., Byrt, D., & Heap, P. (1985). Effect of enzootic pneumonia of pigs on growth performance. *Australian Veterinary Journal*, *62*(1), 13–18. https://doi.org/10.1111/j.1751-0813.1985.tb06032.x
43. Quezada-Fraide, E. A., Peñuelas-Rivas, C. G., Moysén-Albarrán, F. S., Trujillo-Ortega, M. E., & Martínez-Castañeda, F. E. (2021). Productive performance and costs of swine farms with different PRRS virus vaccination protocols. *Revista Mexicana De Ciencias Pecuarias*, *12*(1), 205–216. https://doi.org/10.22319/RMCP.V12I1.5377
44. Ramirez, C. R., Harding, A. L., Forteguerri, E. B. R., Aldridge, B. M., & Lowe, J. F. (2015). Limited efficacy of antimicrobial metaphylaxis in finishing pigs: A randomized clinical trial. *Preventive Veterinary Medicine*, *121*(1–2), 176–178. https://doi.org/10.1016/j.prevetmed.2015.06.002
45. Rapp-Gabrielson, V., Hoover, T., Sornsen, S., Kesl, L., Taylor, L., Jolie, R., Runnels, P., Weigel, D., Yu, S., Opriessnig, T., Ruebling-Jass, K., Strait, E., & Halbur, P. (2008). Effects of *Mycoplasma hyopneumoniae* vaccination in pigs co-infected with *M. hyopneumoniae* and porcine circovirus type 2. *Journal of Swine Health and Production*, *16*(1), 16–26.
46. Renken, C., Nathues, C., Swam, H., Fiebig, K., Weiss, C., Eddicks, M., Ritzmann, M., & Nathues, H. (2021). Application of an economic calculator to determine the cost of porcine reproductive and respiratory syndrome at farm-level in 21 pig herds in Germany. *Porcine Health Management*, *7*(1), 3. https://doi.org/10.1186/s40813-020-00183-x
47. Schaefer, N., & Morrison, R. (2007). Effect on total pigs weaned of herd closure for elimination of porcine reproductive and respiratory syndrome virus. *Journal of Swine Health and Production*, *15*(3), 152–155.
48. Silva, G. S., Yeske, P., Morrison, R. B., & Linhares, D. C. L. (2019). Benefit-cost analysis to estimate the payback time and the economic value of two *Mycoplasma hyopneumoniae* elimination methods in breeding herds. *Preventive Veterinary Medicine*, *168*, 95–102. https://doi.org/10.1016/j.prevetmed.2019.04.008
49. Stipkovits, L., Laky, Z., Abonyi, T., Siugzdaite, J., & Szabó, I. (2003). Reduction of economic losses caused by mycoplasmal pneumonia of pigs by vaccination with Respisure and by Tiamutin treatment. *Acta Veterinaria Hungarica*, *51*(3), 259–271. https://doi.org/10.1556/AVet.51.2003.3.2
50. Stygar, A. H., Niemi, J. K., Oliviero, C., Laurila, T., & Heinonen, M. (2016). Economic value of mitigating *Actinobacillus pleuropneumoniae* infections in pig fattening herds. *Agricultural Systems*, *144*, 113–121. https://doi.org/10.1016/j.agsy.2016.02.005
51. Thomann, B., Rushton, J., Schuepbach-Regula, G., & Nathues, H. (2020). Modeling economic effects of vaccination against porcine reproductive and respiratory syndrome: impact of vaccination effectiveness, vaccine price, and vaccination coverage. *Frontiers in Veterinary Science*, *7*, 500. https://doi.org/10.3389/fvets.2020.00500
52. Trevisan, G., Robbins, R., Angulo, J., Dufresne, L., Lopez, W., Macedo, N., & Linhares, D. (2020). Relationship between weekly porcine reproductive and respiratory syndrome virus exposure in breeding herds and subsequent viral shedding and mortality in the nursery. *Journal of Swine Health and Production*, *28*(5), 244–253.
53. Trevisi, P., Amatucci, L., Ruggeri, R., Romanelli, C., Sandri, G., Luise, D., Canali, M., & Bosi, P. (2022). Pattern of antibiotic consumption in two Italian production chains differing by the endemic status for porcine reproductive and respiratory syndrome. *Frontiers in Veterinary Science*, *9*, 840716. https://doi.org/10.3389/fvets.2022.840716
54. Valdes-Donoso, P., & Jarvis, L. S. (2022). Combining epidemiology and economics to assess control of a viral endemic animal disease: porcine reproductive and respiratory syndrome (PRRS). *PLoS ONE*, *17*(9), e0274382. https://doi.org/10.1371/journal.pone.0274382
55. Young, M. G., Cunningham, G. L., & Sanford, S. E. (2011). Circovirus vaccination in pigs with subclinical porcine circovirus type 2 infection complicated by ileitis. *Journal of Swine Health and Production*, *19*(3), 175–180.
56. Zhang, A., Young, J. R., Suon, S., Ashley, K., Windsor, P. A., & Bush, R. D. (2017). Investigating the financial impact of porcine reproductive and respiratory syndrome on smallholder pig farmers in Cambodia. *Tropical Animal Health and Production*, *49*(4), 791–806. https://doi.org/10.1007/s11250-017-1264-1
57. Zhang, H., Kono, H., & Kubota, S. (2014). An integrated epidemiological and economic analysis of vaccination against highly pathogenic porcine reproductive and respiratory syndrome (PRRS) in Thua Thien Hue province, Vietnam. *Asian-Australasian Journal of Animal Sciences*, *27*(10), 1499–1512. https://doi.org/10.5713/ajas.2014.14060
58. Zhang, Z., Li, Z., Li, H., Yang, S., Ren, F., Bian, T., Sun, L., Zhou, B., Zhou, L., & Qu, X. (2022). The economic impact of porcine reproductive and respiratory syndrome outbreak in four Chinese farms: Based on cost and revenue analysis. *Frontiers in Veterinary Science*, *9*, 1024720. https://doi.org/10.3389/fvets.2022.1024720
